# Prevalence and risk factors of prolonged corrected QT interval in general Chinese population

**DOI:** 10.1186/s12872-019-1244-7

**Published:** 2019-11-29

**Authors:** Qun Ma, Zhao Li, Xiaofan Guo, Liang Guo, Shasha Yu, Hongmei Yang, Lu Zou, Liqiang Zheng, Guowei Pan, Yonghong Zhang, Yingxian Sun

**Affiliations:** 1 Department of Cardiology, the First Hospital of China Medical University, 155 Nanjing North Street, Heping District, Shenyang, 110001 People’s Republic of China; 2 Department of Clinical Epidemiology, Library, Shengjing Hospital of China Medical University, Shenyang, Liaoning People’s Republic of China; 3 Department of Prevention of Chronic Non-communicable Diseases, Center for Disease Prevention and Control of Liaoning Province, Shenyang, Liaoning People’s Republic of China; 4 Department of Epidemiology, School of Public Health, Medical College of Soochow University, Suzhou, Jiangsu People’s Republic of China

**Keywords:** Long QT syndrome, Prevalence, Risk factors

## Abstract

**Background:**

Corrected QT (QTc) interval has been correlated with total and CVD mortality. Although much is known about the relation between prolonged QTc interval and clinical outcome, there is no information on the prevalence and specific risk factors of QTc prolongation in general Chinese population. We evaluated the prevalence of prolonged QTc interval and its risk factors in general Chinese population, aiming to fill in the gaps in the literature and provide evidence for potential CVD risk prediction and disease burden estimate in community.

**Methods:**

A population-based survey was conducted on 11,209 participants over the age of 35 in rural areas of Liaoning Province from 2012 to 2013. Twelve-lead ECGs and automatic analysis were performed on all participants. Logistic regression adjustments were made by using the Bazett’s formula to correlate specific risk factors with prolonged QTc intervals (> 440 ms) for potential confounders.

**Results:**

The overall prevalence of prolonged QTc interval was 31.6%. The prevalence increased significantly with age (24.1% among those aged 35–44 years; 28.3%, 45–54 years; 35.2%, 55–64 years; 43.4%, ≥65 years, *P* < 0.001). Participants with a history of CVD had a higher prevalence of QTc prolongation (40.7% vs. 30.0%). In the fully adjusted logistic regress model, older age, abdominal obesity, hypertension, diabetes, hypokalemia and any medicine used in the past two weeks were associated independently with increased risk for prolonged QTc interval (All *P* < 0.05). We found no significant differences between general obesity, hypocalcemia and hypomagnesemia with prolongation of QTc interval. Female sex showed opposite results after applying clinical diagnostic criteria, and high physical activity could reduce the risk of prolonged QTc interval.

**Conclusions:**

The prevalence of prolonged QTc interval was relatively high in general Chinese population and listed relevant factors, which would help identify patients at risk in pre-clinical prevention and provide evidence for estimating potential CVD burden and making management strategies in community.

## Background

The QT interval on the surface electrocardiogram (ECG) indicates the duration from the beginning of ventricular depolarization to the end of ventricular repolarization. Prolonged heart rate-corrected QT (QTc) interval has been correlated with many adverse cardiovascular outcomes including arrhythmia [1], coronary heart disease [2, 3], sudden death [4] and mortality [5]. Since ECG is a noninvasive and simple test, prolonged QTc interval can be used as a rapid objective method for targeting the population with high CVD risk in clinical practice.

The prevalence of prolonged QTc interval has already been reported in patients with different diseases, such as diabetes (25.6% vs. 7.6%, type 1 diabetes vs. control) [6, 7], HIV infection (19.8%) [8] and coronary heart disease (17.2%) [9], from hospital settings. There is limited information on the prevalence of QTc interval prolongation in general populations. According to the Third National Health and Nutrition Examination Survey (NHANES III), there were 270 and 259 of 4053 men and 4314 women, respectively, had QTc interval prolongation between 1988 and 1994 [10].

Prolonged QTc interval is a result of a complex interplay between genetic and environmental factors [11, 12]. As rapid changes of the pattern of cardiometabolic disease and lifestyle habits, the prevalence and specific risk factors of prolonged QTc interval might be different from decades ago. However, no study has described the epidemiology of QTc interval prolongation in general population from China, recent or not. Using a large, contemporary general Chinese population, we evaluated the prevalence of QTc interval prolongation and its risk factors, aiming to fill in the gaps in the literature and provide evidence for potential CVD risk prediction and disease burden estimate in community.

## Methods

### Study population

Methods of this study has already been published elsewhere [13]. A representative population aged ≥35 years was chosen to exhibit the prevalence of cardiovascular risk factors from January 2012 to August 2013 in rural areas of Liaoning Province, which is situated in the northeast of China. The study used a stratified multi-stage cluster sampling method. In the first phase, three counties (Dawa, Liaoyang, and Zhangwu County) were chosen from the eastern, northern and southern parts of Liaoning province. In the second phase, we selected randomly a town from each county (a total of 3 towns). In the third phase, 8–10 rural villages were selected randomly from each town (a total of 26 rural villages). And this study excluded participants with pregnancy, malignant tumor and mental disorder. All qualified permanent residents aged above 35 years in each village were invited to participate in the research (a total of 14,016 participants). Among those participants, 11,956 participants agreed and completed the research And the response rate was 85.3%. This study was reviewed and approved by the Ethics Committee of China Medical University (Shenyang, China). All procedures were carried out according to the ethics committee standards. All participants’ written consent should be obtained after they had been told and explained about the objectives, benefits, medical items and confidentiality agreement of personal information. Besides, the Ethics Committee has also approved to include the illiterate population. And we have obtained the written consent of all participants themselves or their authorized proxies. We used the baseline data in this report and only participants with complete research information were included, which making a final sample size of 11,209 (5106 males and 6103 females).

### Data collection and measurements

Data collection, measurement methods and the questionnaires of this study have already been published and described previously [14–16]. Cardiologists and trained nurses collected the primary data at a single clinic visit by using standard questionnaires through face-to-face interviews. We invited all qualified investigators to participate in organized training before the investigation. The training content included the purpose of this study, how to conduct questionnaires, the standard measurement methods, the importance of standardization, and the research procedures. After this training, we evaluated a rigorous test, and only those who got a perfect score in the test could become an investigator. Our inspectors received further instructions and support during the data collection process. And there was a central steering committee and a quality control subcommittee to monitor study progress at different stages.

Data of demographic characteristics, family income, lifestyle risk factors, history of heart disease and any medicine used in the past two weeks were obtained through standardized questionnaire interviews. Educational level was classified into 3 groups: primary school or below, middle school and high school or above. Family income was divided into 3 groups: ≤5000, 5000–20,000 and > 20,000 CNY/year. Physical activity was divided into occupational physical activity and leisure physical activity. A detailed description of the method has ever been described elsewhere [17]. Occupational and leisure physical activities were combined and reclassified into three categories: (1) low: reported subjects with mild occupational and leisure physical activity; (2) moderate: reported subjects with moderate or high levels of occupational or leisure physical activity; (3) high: reported subjects with moderate or high levels of occupational and leisure physical activity.

According to the American Heart Association protocol, after at least 5 min of rest, blood pressure (BP) was measured three times every 2 min using a standardized automatic electronic sphygmomanometer (HEM-907; Omron), which had been verified according to the protocol of British Hypertension Society [18]. The participants were told to avoid exercise and caffeinated beverages for at least 30 min before the measurement. During the measurement, the participants sat with the arm supported at the same height of the heart. The average of the three BP values was calculated and used for further analysis.

Height and weight were measured to the nearest 0.1 cm and 0.1 kg, respectively. During this period, the participants wore lightweight clothes and no shoes. Body mass index (BMI) was calculated as body weight in kilograms divided by the square of the body height in meters. Waist circumference (WC) was measured at the level of umbilicus with inelastic tapeline (accurate to 0.1 cm), during which the participants stood at the end of normal expiration.

Fasting blood samples were collected in the morning after all participants had fasted for at least 12 h. All blood samples were taken from the antecubital vein by using a BD Vacutainer tube containing EDTA (Becton, Dickinson and Co., Franklin Lakes, NJ, USA). The serum was then separated from whole blood through the centrifuge, and the serum samples were frozen at − 20 °C for further tests in a certified central laboratory. Total cholesterol (TC), triglyceride (TG), low-density lipoprotein cholesterol (LDL-C), high-density lipoprotein cholesterol (HDL-C), serum potassium, serum calcium, serum magnesium, fasting plasma glucose (FPG), and other routine blood biochemical indexes were analyzed by enzymatic method using an automatic analyzer (Olympus AU640 Auto-Analyzer; Olympus Corp., Kobe, Japan). In addition, all laboratory devices have been calibrated and samples were duplicated by blind method.

A MAC 5500 (GE Healthcare; Little Chalfont, Buckinghamshire, UK) was used by well-trained cardiologists to perform a 12-lead ECGs (rest 10s) for all participants and the results was analyzed automatically through the MUSE Cardiology Information System, version 7.0.0 (GE Healthcare). And Bazett’s formula was then used to correct QT intervals for heart rate [19].

### Definitions

Hypertension was defined as SBP ≥ 140 mmHg and/or DBP ≥ 90 mmHg and/or use of antihypertensive medicines according to the JNC-7 report [20]. According to the recommendations from experts of the WHO for Asians, general obesity was defined as BMI ≥ 28 kg/m^2^ [21]. Abdominal obesity was defined as WC ≥ 102 cm for men and WC ≥ 88 cm for women [22]. Dyslipidemia was diagnosed according to the criteria established by National Cholesterol Education Program-Third Adult Treatment Panel (ATP III) [23]: TC ≥ 6.21 mmol/L (240 mg/dL) for high TC, TG ≥ 2.26 mmol/L (200 mg/dL) for high TG, LDL-C ≥ 4.16 mmol/L (160 mg/dL) for high LDL-C and HDL-C < 1.03 mmol/L (40 mg/dL) for low HDL-C. Diabetes mellitus was defined according to the WHO criteria [24]: FPG ≥ 7 mmol/L (126 mg/dL) and/or receiving diabetes treatment. According to recognized criteria, the QT interval corrected by the Bazett’s formula was considered to have been prolonged if it exceeds 440 ms [25], and we set 450 ms for men and 470 ms for women to further adjust gender for specific prolonged QTc interval.

### Statistical analysis

Descriptive statistics of all study variables had been calculated, including categorical variables (expressed as numbers and ratios) and continuous variables (expressed as mean values and standard deviations). Non-parameter test or an appropriate χ2-test were used to evaluate the differences among these categories. Multivariate logistic regression analysis was adopted to calculate the possible risk factors (age, gender, race, family income, physical activity, education, current smoking status, current drinking status, obesity, hypertension, diabetes, high TC, high TG, high LDL-C, low HDL-C, serum electrolytes and medication) for QTc interval prolongation with odds ratios (ORs) and corresponding 95% confidence intervals (CIs). All statistical analyses were conducted using SPSS version 23.0 software, and *P* values less than0.05 were considered as statistically significant.

## Results

### Baseline characteristics of study population

The present study included 5106 males and 6103 females, with an average age of 54 (SD 11) years. Table 1 lists the differences of characteristics in research population based on QTc grouping. Older participants were more likely to have prolonged QTc interval (> 440 ms). A significantly lower proportion of prolonged QTc interval was observed among males. Participants with prolonged QTc interval had higher levels of cardiometabolic indexes such as SBP, DBP, TC, TG, LDL-C and FPG (all *P* < 0.05), while current smokers or drinkers were more common among those participants with normal QTc interval.

**Table 1:** Differences of characteristics in study population based on QTc grouping (*N* = 11,209)

Variables	QTc ≤ 440 ms (*n* = 7666)	QTc > 440 ms (*n* = 3543)	*P*-value
Age (year)	53 ± 10	56 ± 11	< 0.001^*^
Male gender	4063 (53.0)	1043 (29.4)	< 0.001^*^
Race of Han	7250 (94.6)	3380 (95.4)	0.066
Current smoking status	2915 (38.0)	1028 (29.0)	< 0.001^*^
Current drinking status	1981 (25.8)	520 (14.7)	< 0.001^*^
Education			< 0.001^*^
Primary school or below	3603 (47.0)	1987 (56.1)	
Middle school	3326 (43.4)	1254 (35.4)	
High school or above	737 (9.6)	302 (8.5)	
Physical activity			< 0.001^*^
Low	2013 (26.3)	1278 (36.1)	
Moderate	5210 (68.0)	2076 (58.6)	
High	443 (5.8)	189 (5.3)	
Family income (CNY/year)			< 0.001^*^
≤ 5000	876 (11.4)	513 (14.5)	
5000–20,000	4179 (54.5)	1952 (55.1)	
> 20,000	2611 (34.1)	1078 (30.4)	
SBP (mmHg)	138.9 ± 22.1	148.0 ± 25.2	< 0.001^*^
DBP (mmHg)	80.9 ± 11.1	84.4 ± 12.7	< 0.001^*^
BMI (kg/m^2^)	24.6 ± 3.6	25.2 ± 3.8	< 0.001^*^
WC (cm)	81.9 ± 9.7	83.6 ± 9.9	< 0.001^*^
TC (mmol/L)	5.2 ± 1.1	5.4 ± 1.2	< 0.001^*^
TG (mmol/L)	1.6 ± 1.4	1.8 ± 1.7	< 0.001^*^
LDL-C (mmol/L)	2.9 ± 0.8	3.0 ± 0.9	< 0.001^*^
HDL-C (mmol/L)	1.4 ± 0.4	1.4 ± 0.4	0.014^*^
FPG (mmol/L)	5.8 ± 1.4	6.2 ± 2.1	< 0.001^*^
Calcium (mmol/L)	2.3 ± 0.1	2.3 ± 0.1	< 0.001^*^
Potassium (mmol/L)	4.2 ± 0.3	4.1 ± 0.4	< 0.001^*^
Magnesium (mmol/L)	0.8 ± 0.1	0.8 ± 0.1	0.119
History of heart disease^a^	647 (8.4)	451 (12.7)	< 0.001^*^
Medication used^b^	3806 (49.6)	2122 (59.9)	< 0.001^*^

Data are expressed as the mean ± SD or as n (%)

Abbreviations: BMI, body mass index; CNY, China Yuan (1CNY = 0.15 USD); DBP, diastolic blood pressure; FPG, fasting plasma glucose; HDL-C, high-density lipoprotein cholesterol; LDL-C, low-density lipoprotein cholesterol; QTc, corrected QT; SBP, systolic blood pressure; TC, total cholesterol; TG, triglyceride; WC, waist circumference

*P < 0.05 for the significance of difference of variables between prolonged QTc interval and QTc ≤ 440 ms.

^a^Including coronary heart disease, arrythmia and heart failure

^b^Indicating any self-reported medication used in the past two weeks

### Prevalence of prolonged QTc interval

**Fig. 1:**
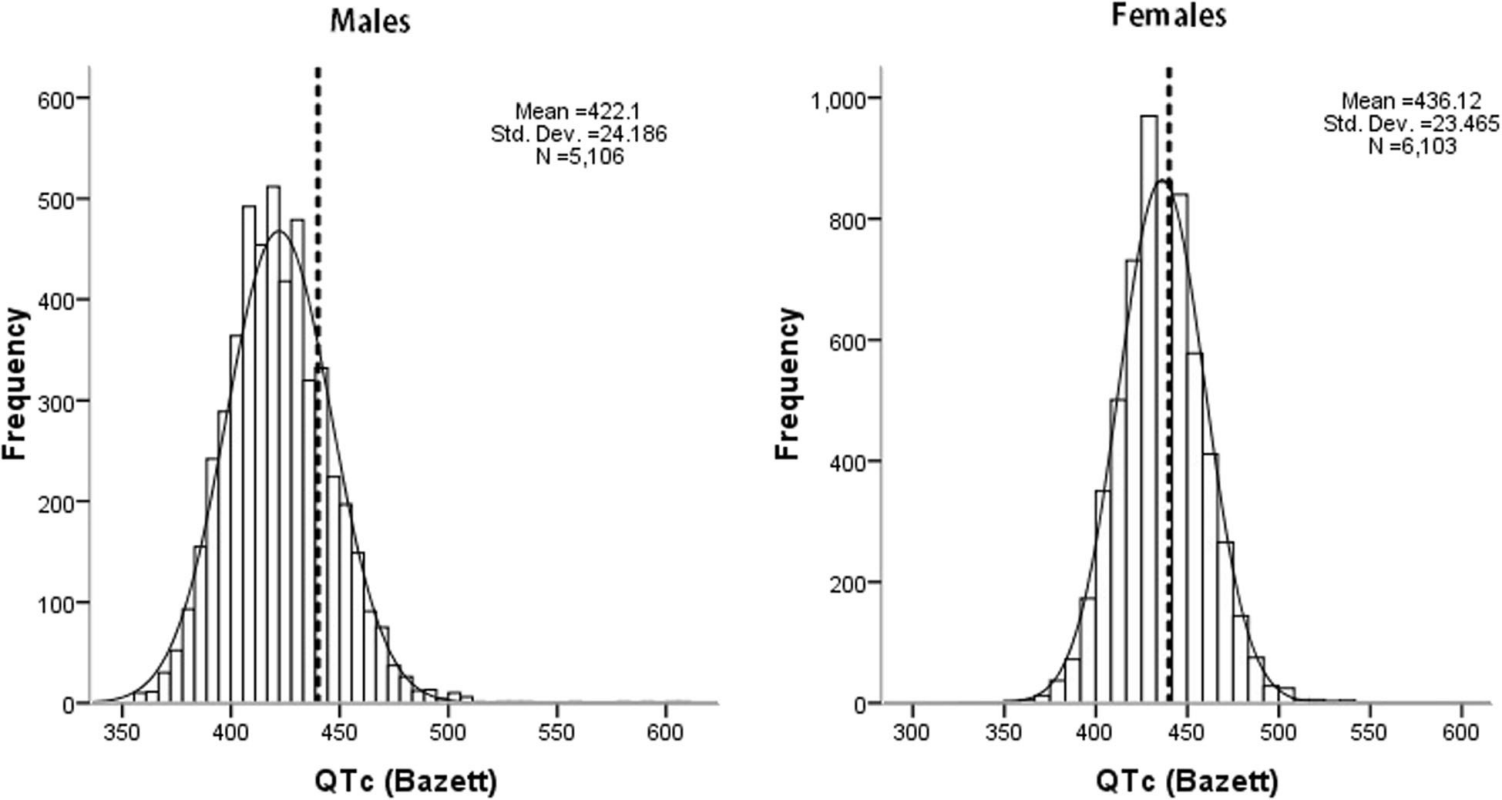
Distribution of QTc interval of the population by sex

**Fig. 2:**
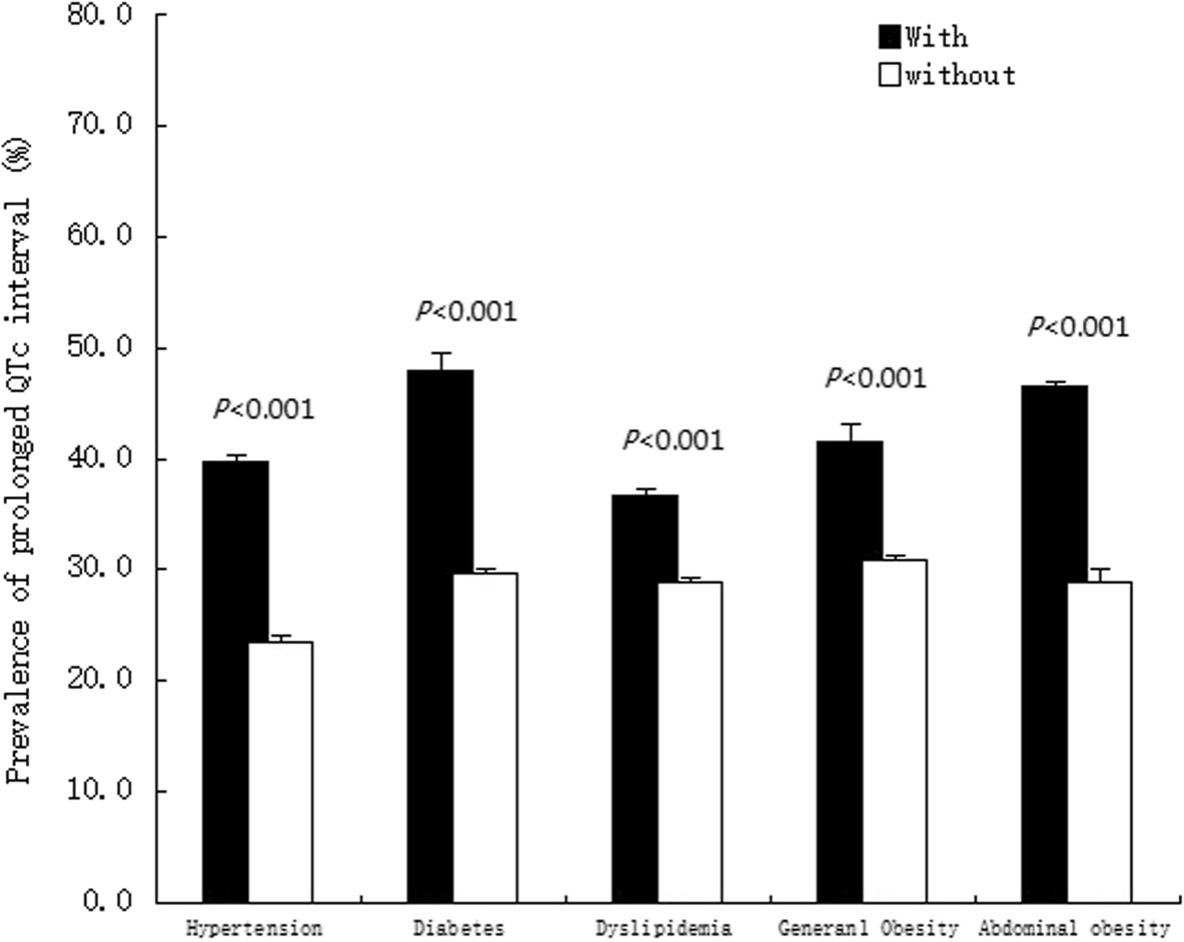
Prevalence of prolonged QTc interval with and without major clinical comorbidities

Table 2 shows the distribution of QTc interval according to age and sex. The overall prevalence of QTc prolongation was 31.6%. The mean QTc interval was significantly longer among females than males (436.1 ± 23.5 vs. 422.1 ± 24.2 ms, respectively, *P* < 0.001, Fig. 1), which was observed across all the age groups. The prevalence of prolonged QTc interval was significantly correlated with age (24.1% in the 35–44 age group; 28.3% in the 45–54 age group; 35.2% in the 55–64 age group; 43.4% in the ≥65 age group, *P* < 0.001). The prevalence of QTc interval prolongation was also significantly higher among female participants in all four age groups. While after adjusting specific prolonged QTc interval for sex, this result reverse. Figure 2 shows the prevalence of prolonged QTc interval in different clinical comorbidities. A relatively higher prevalence was observed among those participants with either of the major comorbidities, including hypertension, diabetes, dyslipidemia, general and abdominal obesity (all *P* < 0.001). In addition, the prevalence of prolonged QTc interval was higher among the participants with CVD history (40.7% vs. 30.0%, 671/1650 vs. 2871/9559).

**Table 2:** Distribution of QTc interval in the included population by age and sex

Age (year)	Sample size	Mean QTc (ms)		Prevalence of prolonged QTc		Prevalence of prolonged QTc adjusted for sex	
		Male	Female	Male	Female	Male> 450	Female> 470
35–44	2699	415.4 ± 21.7	431.6 ± 23.2	149 (12.6)	502 (33.0)	80 (6.8)	62 (4.1)
45–54	3481	419.3 ± 23.3	434.4 ± 22.1	256 (16.7)	730 (37.5)	140 (9.1)	106 (5.4)
55–64	3371	424.7 ± 24.6	438.6 ± 23.6	358 (22.9)	829 (45.8)	191 (12.2)	135 (7.5)
≥65	1658	431.8 ± 24.5	443.3 ± 24.5	280 (33.5)	439 (53.3)	180 (21.6)	92 (11.2)

Data are expressed as the mean ± SD or as n (%)

Abbreviations: QTc, corrected QT

### Factors associated with prolonged QTc interval

Table 3 shows the multivariable logistic regression analysis of risk factors associated with prolonged QTc interval. The risk of prolonged QTc interval increased by 22.8% for every 10 years of age (OR: 1.228, 95%CI: 1.168 to 1.290, *P* < 0.001). Prolonged QTc interval (QTc > 440 ms) was found in 1043 (29.3%) and 2500 (70.6%) of male and female, which is showed in Table 1. Compared with males, the risk of females QTc interval prolongation increased about 3 times (OR: 2.878, 95%CI: 2.564 to 3.229, *P* < 0.001). Participants with hypertension and diabetes had increased by 83.8 and 59.3% risks respectively for prolonged QTc interval (OR: 1.838, 95%CI: 1.675 to 2.016; OR: 1.593, 95%CI: 1.391 to 1.824 respectively, both *P* < 0.001). Abdominal obesity, high TG and low potassium level were also significantly associated with prolonged QTc interval, which increased by 15.6, 24.1 and 163.6% risks respectively for prolonged QTc interval (OR: 1.156, 95%CI: 1.010 to 1.324, *P* = 0.035; OR: 1.241, 95%CI: 1.104 to 1.395, *P* < 0.001; OR: 2.636, 95%CI: 1.806 to 3.846, *P* < 0.001). Recent use of any medication achieved statistical significance for 13.1% higher risk of prolonged QTc interval (OR: 1.131, 95%CI: 1.036 to 1.234, *P* = 0.006). To further eliminate the interference of self-reported medication use, we performed a sensitivity analysis excluding the participants on medication (Table 4). Age, female sex, abdominal obesity, hypertension, diabetes and hypokalemia were still risk factors for prolonged QTc interval, except for high TG. Specifically, the risk increased by 21.6% for every 10 years of age (OR: 1.216, 95%CI: 1.127 to 1.311, *P* < 0.001). And female sex, abdominal obesity, hypertension, diabetes and hypokalemia increased by 199.5, 26.4, 93.3, 85.6 and 122.4% respectively (OR: 2.995, 95%CI: 2.502 to 3.584, *P* < 0.001; OR: 1.264, 95%CI: 1.019 to 1.567, *P* = 0.033; OR: 1.933, 95%CI: 1.680 to 2.224, *P* < 0.001; OR: 1.856, 95%CI: 1.469 to 2.346, *P* < 0.001; OR: 2.224, 95%CI: 1.118 to 4.426, *P* = 0.023). Furthermore, compared to low physical activity, high physical activity reduced to 70.2% risk for prolonged QTc interval (OR: 0.702, 95%CI: 0.525 to 0.939, *P* = 0.017). While after setting 450 ms for men and 470 ms for women, which is the clinical diagnosis criteria for long QT syndrome (Prolonged QTc interval was found in 591 (59.9%) and 395 (40.1%) of male and female), female sex might be a protective factor among those population, which the risk reduced to 49.7% (OR: 0.497, 95%CI: 0.416 to 0.594, *P* < 0.001) in Table 5. And high physical activity remained a protective factor for reducing the risk to 65.2% (OR: 0.652, 95%CI: 0.462 to 0.921, *P* = 0.015).

**Table 3:** Multivariate logistic regression analyses of prolonged QTc interval and associated factors

Variables	ORs	95% CIs	*P*-value
Age (per 10 year increase)	1.228	1.168–1.290	< 0.001^*^
Female gender	2.878	2.564–3.229	< 0.001^*^
Race of Han^a^	1.196	0.981–1.458	0.076
Current smoking status	1.075	0.970–1.191	0.166
Current drinking status	0.938	0.821–1.071	0.344
Education			
Primary school or below	1.000 (reference)		
Middle school	1.075	0.974–1.186	0.151
High school or above	1.157	0.986–1.357	0.073
Physical activity			
Low	1.000 (reference)		
Moderate	0.925	0.840–1.018	0.11
High	0.832	0.685–1.011	0.064
Family income (CNY/year)			
≤ 5000	1.000 (reference)		
5000–20,000	0.931	0.815–1.064	0.294
> 20,000	0.926	0.799–1.072	0.302
General obesity	1.043	0.916–1.187	0.528
Abdominal obesity	1.156	1.010–1.324	0.035^*^
Hypertension	1.838	1.675–2.016	< 0.001^*^
Diabetes	1.593	1.391–1.824	< 0.001^*^
High TC	0.977	0.847–1.127	0.747
High TG	1.241	1.104–1.395	0.001^*^
High LDL-C	1.112	0.919–1.347	0.276
Low HDL-C	1.119	0.980–1.278	0.096
Calcium (mmol/L) < 1.875	0.667	0.178–2.497	0.547
Potassium (mmol/L) < 3.5	2.636	1.806–3.846	< 0.001^*^
Magnesium (mmol/L) < 0.7	0.746	0.200–2.789	0.664
History of heart disease^b^	1.065	0.927–1.223	0.375
Medication used^c^	1.131	1.036–1.234	0.006^*^

*P < 0.05 for the independent association between prolonged QTc interval and each factor after adjusting for the remaining factors

Abbreviations: CNY, China Yuan (1CNY = 0.15 USD); HDL-C, high-density lipoprotein cholesterol; LDL-C, low-density lipoprotein cholesterol; TC, total cholesterol; TG, triglyceride; OR, odds ratio; QTc, corrected QT; 95% CI, 95% confidence interval

^a^Compared with other ethnic minorities in China, such as Mongol and Manchu

^b^Including coronary heart disease, arrythmia and heart failure

^c^Indicating any self-reported medication used in the past two weeks

**Table 4:** Sensitivity analysis of prolonged QTc interval and associated factors after exclusion of participants on medication in the past two weeks in multiple logistic regression (*n* = 5281)

Variables	ORs	95% CIs	*P*-value
Age (per 10 year increase)	1.216	1.127–1.311	< 0.001^*^
Female gender	2.995	2.502–3.584	< 0.001^*^
Race of Han^a^	1.260	0.944–1.682	0.117
Current smoking status	0.948	0.808–1.112	0.511
Current drinking status	1.020	0.842–1.237	0.836
Education			
Primary school or below	1.000 (reference)		
Middle school	1.086	0.936–1.261	0.276
High school or above	1.294	1.029–1.626	0.027^*^
Physical activity			
Low	1.000 (reference)		
Moderate	0.879	0.756–1.023	0.096
High	0.702	0.525–0.939	0.017^*^
Family income (CNY/year)			
≤ 5000	1.000 (reference)		
5000–20,000	1.024	0.824–1.273	0.828
> 20,000	1.105	0.876–1.393	0.401
General obesity	1.089	0.891–1.330	0.407
Abdominal obesity	1.264	1.019–1.567	0.033^*^
Hypertension	1.933	1.680–2.224	< 0.001^*^
Diabetes	1.856	1.469–2.346	< 0.001^*^
High TC	0.987	0.793–1.229	0.909
High TG	1.172	0.973–1.413	0.094
High LDL-C	1.142	0.843–1.547	0.391
Low HDL-C	1.214	0.987–1.492	0.066
Calcium (mmol/L) < 1.875	0.603	0.071–5.106	0.642
Potassium (mmol/L) < 3.5	2.224	1.118–4.426	0.023^*^
Magnesium (mmol/L) < 0.7	1.893	0.323–11.098	0.479
History of heart disease^b^	0.916	0.697–1.204	0.529

*P < 0.05 for the independent association between prolonged QTc interval and each factor after adjusting for the remaining factors

Abbreviations: CNY, China Yuan (1CNY = 0.15 USD); HDL-C, high-density lipoprotein cholesterol; LDL-C, low-density lipoprotein cholesterol; TC, total cholesterol; TG, triglyceride; OR, odds ratio; QTc, corrected QT; 95% CI, 95% confidence interval

^a^Compared with other ethnic minorities in China, such as Mongol and Manchu

^b^Including coronary heart disease, arrythmia and heart failure

**Table 5:** Multivariate logistic regression analyses of prolonged QTc interval and associated factors after adjusting the clinical diagnosis criteria for gender

Variables	ORs	95% CIs	*P*-value
Age (per 10 year increase)	1.257	1.164–1.357	< 0.001^*^
Female gender	0.497	0.416–0.594	< 0.001^*^
Race of Han^a^	0.827	0.617–1.109	0.205
Current smoking status	1.015	0.870–1.184	0.847
Current drinking status	1.085	0.905–1.300	0.379
Education			
Primary school or below	1.000 (reference)		
Middle school	1.010	0.862–1.184	0.900
High school or above	1.014	0.784–1.312	0.916
Physical activity			
Low	1.000 (reference)		
Moderate	0.896	0.768–1.046	0.164
High	0.652	0.462–0.921	0.015^*^
Family income (CNY/year)			
≤ 5000	1.000 (reference)		
5000–20,000	0.883	0.728–1.073	0.211
> 20,000	0.848	0.680–1.059	0.146
General obesity	1.097	0.842–1.430	0.493
Abdominal obesity	1.191	0.950–1.493	0.130
Hypertension	2.357	2.001–2.775	< 0.001^*^
Diabetes	1.638	1.362–1.970	< 0.001^*^
High TC	0.865	0.689–1.087	0.214
High TG	1.231	1.033–1.467	0.020^*^
High LDL-C	1.111	0.826–1.495	0.486
Low HDL-C	1.143	0.937–1.394	0.187
Calcium (mmol/L) < 1.875	0.830	0.103–6.713	0.861
Potassium (mmol/L) < 3.5	1.957	1.230–3.114	0.005^*^
Magnesium (mmol/L) < 0.7	0.590	0.072–4.860	0.624
History of heart disease^b^	1.375	1.126–1.678	0.002^*^
Medication used^c^	1.253	1.085–1.448	0.002^*^

*P < 0.05 for the independent association between prolonged QTc interval and each factor after adjusting for the remaining factors

Abbreviations: QTc, corrected QT; OR, odds ratio; 95% CI, 95% confidence interval; CNY, China Yuan (1CNY = 0.15 USD);

TC, total cholesterol; TG, triglyceride; LDL-C, low-density lipoprotein cholesterol; HDL-C, high-density lipoprotein cholesterol

^a^Compared with other ethnic minorities in China, such as Mongol and Manchu

^b^Including coronary heart disease, arrythmia and heart failure

^c^Indicating any self-reported medication used in the past two weeks

## Discussion

In the present study, we reported a relatively higher prevalence of prolonged QTc interval in general Chinese population compared to Hawaii. We found age, abdominal obesity, hypertension, diabetes, hypokalemia and any medicine used in the past two weeks were associated with increased risk for prolongation of QTc interval.

We found the overall prevalence of QTc prolongation was 31.6%, by a 440 ms cut-point for definition using Bazett’s formula, which is the most common method for heart rate adjustment [26]. In a rural Hawaiian population, the prevalence of prolonged QTc interval was found to be 21.2%, using the same criteria [27]. Compared with the baseline characteristics of long QTc interval from a Japanese cohort, the prevalence of which were around 7.5% among males men and 16.5% among females [28], our prevalence were much higher (20.4% for men and 41% for women). In subgroup analyses, the prevalence among patients with diabetes was also higher than that observed in previous studies using similar criteria [7, 29, 30]. Regional and racial differences might contribute to the inconsistent results. Since the patterns of cardiometabolic diseases and lifestyle factors have important influence on the prevalence, the conducted year of the study might also contribute to the discrepancies. Prolonged QTc interval was closely related to increased risks of total death, cardiovascular death, coronary heart disease death, and sudden cardiac death [26]. Therefore, the high prevalence we observed in general Chinese population indicated a potential large burden of CVD prevention in rural China.

We confirmed the impacts of age, diabetes and hypertension on prolonged QTc interval which were consistent with previous findings [6, 8, 27, 29, 31]. As for gender, female sex of the borderline groups (440 ms < QTc < 450 ms for males and 440 ms < QTc < 470 ms for females) were more likely to develop long QT syndrome (QTc > 450 ms for males and QTc > 470 ms for females), which leads to female as a risk factor after setting 440 ms for prolonged QTc interval. Furthermore, after using the clinical diagnosis criteria for long QT syndrome to exclude borderline population, we found female seems to be less likely for long QT syndrome in the population. Although the results were inconsistent, it’s certain that sex hormones might modulate ventricular repolarization and partly attributed to the sex-specific difference on QTc interval, which indicating female seems to have higher QTc interval compared to male among the borderline population [32–34]. The presence of hypertension and diabetes could lead to significant higher risks for prolonged QTc interval, indicating that a screening ECG should be warranted before prescribing medications in these populations.

It has been shown that general obesity is correlated with prolonged QTc interval [35–37]. However, we failed to find a significant correlation between general obesity and prolonged QTc interval. Although the prevalence was higher among participants with general obesity, there was no association observed in multivariable models, which was consistent with previous findings [10, 29]. In contrast, abdominal obesity was an independent risk factor for prolonged QTc, suggesting that visceral obesity played a more important role in the obesity-related risk and prolongation of QTc. We also found a significant correlation between high TG and prolonged QTc interval in the data set including medication use, but not in sensitivity analysis. More researches are expected to confirm this relationship.

The strength of the present study was that we for the first time provided a detailed epidemiologic description of prolonged QTc interval in a general population from China with a large sample size and reflects the differences of the prevalence among hypertension, diabetes, dyslipidemia, general obesity and abdominal obesity. Our study had some limitations that need to be considered. First, instead of using sex-specific tertiles for QTc interval, we chose 440 ms as the definition for prolonged QTc interval for both sexes to include all borderline subgroups, and we set 450 ms for men and 470 ms for women to identify specific long QT syndrome. Besides, we used automatically measured QTc values and Bazett’s formula to further analysis. A pooled analysis found that long QTc interval was significantly associated with total and CVD mortality, using 440 ms as the cut-point for the highest category for both sexes in most of the included studies [26]. Our goal was to capture the risk factors of those participants at even slightly elevated risk. Second, although we adjusted most crucial confounders such as serum electrolytes, there were still other factors, including cardiomyopathy, cirrhosis and genetic determinants, that were not accounted for in this study. And for the physical activity, it is contradictory to interpret high physical activity means protective factor for prolongation of QTc among Tables 3, 4, 5. There is no declared specific recommended physical activity for long-QTc subjects, and some intense physical activities could lead to sudden cardiac death [38, 39]. In general, although it seemed that high physical activity is a protective factor for prolongation of QTc among this population, it may also lead to some occurrences of sudden cardiac death. Besides, this study did not obtain specific monitoring data on physical activity. However, the self-administered questionnaire we collected strengthened the results. Another concern was the medicine history of the participants. We failed to obtain the specific information on QT-prolonging drugs. However, the medication used in the past two weeks we collected included QT-prolonging drugs as well as many other drugs that could also cause prolonged QTc interval, such as antibiotics and some Chinese traditional medicines, which strengthened the results. On the other hand, the result of our sensitivity analysis excluding participants on medication was consistent. Furthermore, our results were based on cross-sectional design, so no causal relationship could be established.

## Conclusions

We found the prevalence of QTc interval prolongation is higher in the general Chinese population, especially those with CVD history. Older age, female sex, abdominal obesity, hypertension, diabetes and hypokalemia were associated with increased risks of prolonged QTc interval. This finding would help identify patients at risk in pre-clinical prevention and provide evidence for estimating potential CVD burden and making management strategies for rural Chinese population.

## Data Availability

The data used and/or analysed during the current research are available. from the corresponding author on reasonable request.

## Abbreviations

BMI: Body mass index
BP: Blood pressure
CIs: Confidence intervals
CVD: Cardiovascular disease
ECG: Electrocardiogram
FPG: Fasting plasma glucose
HDL-C: High-density lipoprotein cholesterol
LDL-C: Low-density lipoprotein cholesterol
ORs: Odds ratios
QTc: Bazett’s corrected QT
TC: Total cholesterol
TG: Triglyceride
WC: Waist circumference
